# Eating Disorder Risk, Healthy Orthorexia, and Orthorexia Nervosa Among Women Following Vegan, Vegetarian, and Omnivorous Dietary Patterns in Türkiye: A Cross-Sectional Study

**DOI:** 10.3390/healthcare14182914

**Published:** 2026-09-09

**Authors:** Funda Işık, Yaşar Nuri Şahin

**Affiliations:** Department of Nutrition and Dietetics, Faculty of Health Sciences, Kastamonu University, 37150 Kastamonu, Türkiye

**Keywords:** plant-based diets, disordered eating, eating attitudes, healthy orthorexia, orthorexia nervosa, food choice motives, dietary patterns

## Abstract

**Highlights:**

**What are the main findings?**

- No statistically significant adjusted association was detected between eating disorder risk and either vegan or vegetarian dietary patterns.
- Higher healthy orthorexia scores were associated with the vegan pattern, whereas the association between orthorexia nervosa and the vegan pattern was nonlinear.

**What are the implications of the main findings?**

- Vegan and vegetarian dietary pattern labels should not be interpreted as indicators of eating pathology; assessment should consider weight- and shape-related concerns, dietary rigidity, distress, and functional impairment.
- Distinguishing healthy orthorexia from orthorexia nervosa may help dietitians and other healthcare professionals differentiate a non-pathological interest in healthy eating from eating patterns characterized by rigidity, distress, or functional impairment.

**Abstract:**

**Background/Objectives:** Food exclusions in vegan and vegetarian diets may complicate the assessment of disordered eating and orthorexia. This study compared eating disorder risk, healthy orthorexia, and orthorexia nervosa across vegan, vegetarian, and omnivorous women and examined their adjusted associations with dietary pattern. **Methods:** This cross-sectional study used convenience sampling to recruit 305 women in Türkiye who self-identified as vegan (*n* = 64), vegetarian (*n* = 71), or omnivorous (*n* = 170). Participants completed the Eating Attitudes Test-26, Teruel Orthorexia Scale, and Food Choice Questionnaire. Associations with vegan and vegetarian patterns were examined using multinomial logistic regression, with omnivorous women as the reference group. Potential nonlinearity in the association between orthorexia nervosa and the vegan versus omnivorous contrast was examined using restricted cubic splines. Secondary exploratory food choice models were corrected for multiple testing. **Results:** Elevated eating disorder risk was not significantly associated with either the vegan or vegetarian pattern in the adjusted model. Higher healthy orthorexia scores were associated with greater odds of following a vegan pattern (adjusted odds ratio (aOR) = 1.177, 95% confidence interval (CI): 1.090–1.270), whereas the association between orthorexia nervosa and the vegan pattern was nonlinear (*p* for nonlinearity = 0.006), with the adjusted probability declining across low-to-moderate scores and the estimates becoming imprecise at higher scores. Neither orthorexia dimension was associated with the vegetarian pattern. In secondary exploratory analyses, weight control motivation was associated with lower odds of vegan (aOR = 0.424, 95% CI: 0.249–0.723) and vegetarian patterns (aOR = 0.610, 95% CI: 0.382–0.975), while familiarity was associated with lower odds of the vegan pattern (aOR = 0.466, 95% CI: 0.296–0.735). **Conclusions:** Healthy orthorexia and orthorexia nervosa showed distinct associations with the vegan pattern. Dietitians and healthcare professionals should not infer eating pathology from dietary pattern labels alone but should consider motivations for food exclusions, weight- and shape-related concerns, dietary rigidity, distress, and functional impairment.

## 1. Introduction

A vegetarian dietary pattern involves abstaining from meat, poultry, and seafood, whereas a vegan dietary pattern additionally excludes all animal-derived foods [1]. Beyond their defining food exclusions, these dietary patterns may form part of an individual’s self-concept and social identity [2], while their adoption may be shaped by health, ethical, and environmental considerations that differ in relative importance between vegans and vegetarians [3]. Examining vegan, vegetarian, and omnivorous dietary patterns as separate groups may provide a clearer understanding of their relationships with eating-related characteristics.

The intentional food exclusions that define vegan and vegetarian dietary patterns do not, in themselves, indicate disordered eating [4]. Nevertheless, studies examining associations between these dietary patterns and disordered eating characteristics have produced mixed findings [5,6,7,8,9]. A systematic review found no consensus regarding these associations, partly because of variation in dietary definitions, study populations, and assessment instruments [4]. More recently, a systematic review and meta-analysis found no significant pooled difference in eating disorder symptom levels between adults following vegan or vegetarian diets and those following omnivorous diets [10]. However, because the available evidence is predominantly cross-sectional, the temporal direction of any observed associations remains unclear.

The possible relationship between vegan or vegetarian dietary patterns and orthorexia nervosa has also received attention in the literature [11]. A recent expert consensus characterized orthorexia nervosa as a pathological preoccupation with healthy eating accompanied by rigidity, distress, or adverse nutritional and psychosocial consequences [12]. In contrast, healthy orthorexia reflects a non-pathological interest in and tendency toward healthy eating [13,14]. The Teruel Orthorexia Scale was developed to assess these related but conceptually distinct dimensions separately [13]. Supporting this distinction, only orthorexia nervosa—not healthy orthorexia—was associated with elevated disordered eating and clinical impairment [14]. Distinguishing between these dimensions is particularly important when assessing individuals following vegan or vegetarian dietary patterns. For dietitians and other healthcare professionals, assessment should focus not on the food exclusions alone but on whether they are accompanied by rigidity, distress, nutritional compromise, or psychosocial impairment [12,14].

Studies comparing orthorexia across dietary patterns have yielded findings that vary according to the construct and assessment instrument used [15,16]. A recent systematic review and meta-analysis found higher orthorexia nervosa symptom levels among adults following vegan or vegetarian diets than among omnivorous adults, although all included studies were cross-sectional and the pooled evidence was limited by substantial heterogeneity and variation in orthorexia assessment [17]. Studies using bidimensional measures have provided a more differentiated but still inconsistent picture. Şentürk et al. [16] reported higher healthy orthorexia among vegan participants and higher orthorexia nervosa among both vegan and vegetarian participants. In contrast, Albery et al. [18] found higher healthy orthorexia scores among vegan and vegetarian participants than among omnivores but no dietary group differences in orthorexia nervosa. In a sample from Türkiye, Bellikci Koyu et al. [19] reported higher healthy orthorexia scores among vegetarians than among omnivores. Although orthorexia nervosa scores did not differ between these groups in the unadjusted comparison, higher orthorexia nervosa scores were associated with lower odds of following a vegetarian dietary pattern in the adjusted model. Together, these findings reinforce the importance of examining healthy orthorexia and orthorexia nervosa separately.

Food choice motives may provide complementary behavioral context for differences among dietary patterns. Previous studies comparing dietary patterns have reported differences in food choice motives, including health, weight control, familiarity, and ethical concern, although the direction and magnitude of these differences have varied across studies [15,20,21]. Given the primary focus on eating disorder risk, healthy orthorexia, and orthorexia nervosa, food choice motives were examined as secondary exploratory correlates of dietary pattern.

Existing findings regarding eating disorder risk and the two dimensions of orthorexia across dietary patterns remain difficult to reconcile because of differences in dietary group definitions, assessment instruments, and analytical approaches. Therefore, the primary objective of this study was to compare eating disorder risk, healthy orthorexia, and orthorexia nervosa across vegan, vegetarian, and omnivorous women and to examine whether these characteristics were independently associated with following a vegan or vegetarian rather than an omnivorous dietary pattern after adjustment for relevant covariates. As a secondary objective, food choice motives were compared across the three dietary patterns, and their adjusted associations with following a vegan or vegetarian rather than an omnivorous dietary pattern were explored. Given the mixed evidence, no directional hypotheses were specified for the primary associations.

## 2. Materials and Methods

### 2.1. Study Design and Participants

This cross-sectional online study included women aged ≥ 18 years who resided in Türkiye and self-identified as vegan, vegetarian, or omnivorous. The sample was restricted to women to reduce sex-related heterogeneity in eating disorder symptoms and orthorexia-related characteristics; consequently, the findings are not generalizable to men. Participants were recruited through convenience sampling using social media announcements. Dietary pattern classification was based solely on self-identification and was not independently verified through a detailed assessment of animal-derived food consumption. No formal a priori sample size calculation was performed; all eligible nonduplicate questionnaire submissions received during the predefined data collection period were included. This study was reported in accordance with the STROBE guidelines for cross-sectional studies.

### 2.2. Procedure and Ethical Considerations

Data were collected between April and December 2024 using an online questionnaire administered through Google Forms. Before participation, individuals were informed about the study purpose and procedures, the confidentiality of their responses, and the voluntary nature of participation. Electronic informed consent was obtained before access to the questionnaire. No financial or material incentives were provided, and participants could withdraw before submission by closing the survey.

A total of 312 responses were received. Before analysis, records with identical submission timestamps and response patterns were classified as duplicates. Seven duplicate records were excluded, leaving 305 responses in the final analytical sample. All questionnaire items required for the analyses were configured as mandatory; therefore, the form could not be submitted unless these items had been completed, and no missing values were present in the analytical dataset.

This study was approved by the Kastamonu University Clinical Research Ethics Committee (protocol code: 2023-KAEK-88 and date of approval: 2 August 2023) and conducted in accordance with the Declaration of Helsinki.

### 2.3. Measures

#### 2.3.1. Sociodemographic and Anthropometric Characteristics

Participants reported their age, educational attainment, body weight, and height. Body mass index (BMI) was calculated as weight in kilograms divided by height in meters squared (kg/m^2^). For descriptive analyses, BMI was categorized as underweight (<18.5 kg/m^2^), normal weight (18.5–24.9 kg/m^2^), overweight (25.0–29.9 kg/m^2^), or obesity (≥30.0 kg/m^2^), and age was categorized as 18–24, 25–34, 35–44, or ≥45 years. Age was entered into the regression models as a categorical variable, with 18–24 years as the reference category, whereas BMI was entered as a continuous variable.

#### 2.3.2. Eating Attitudes Test-26

The Eating Attitudes Test-26 (EAT-26) is a 26-item self-report measure of attitudes and behaviors associated with disordered eating [22]. Items were scored according to the standard procedure, with item 26 reverse scored. Higher total scores indicate greater disordered eating symptom levels, and a score of ≥20 was used as a screening indicator of elevated eating disorder risk. The validated Turkish version was used [23].

#### 2.3.3. Teruel Orthorexia Scale

The Teruel Orthorexia Scale assesses healthy orthorexia and orthorexia nervosa as conceptually distinct dimensions [13]. Items are rated on a four-point scale. The validated 16-item Turkish version comprises nine healthy orthorexia items and seven orthorexia nervosa items [24]. Items within each subscale were summed, yielding theoretical score ranges of 0–27 and 0–21. Higher scores indicate higher levels of the corresponding construct.

#### 2.3.4. Food Choice Questionnaire

The 36-item Food Choice Questionnaire assesses nine food choice motives: health, mood, convenience, sensory appeal, natural content, price, weight control, familiarity, and ethical concern [25]. The validated Turkish version was used [26]. Items are rated on a four-point scale, and the mean score for each subscale was calculated, with higher scores indicating greater importance assigned to the corresponding motive.

Internal consistency coefficients for all scales and subscales in the present sample are reported in Supplementary Table S1.

### 2.4. Statistical Analysis

Categorical variables were summarized as frequencies and percentages and compared using Pearson’s chi-square test, with Monte Carlo *p* values used when expected cell count assumptions were not met. Continuous variables were summarized as medians and interquartile ranges and compared using Kruskal–Wallis tests. Cramér’s V and epsilon-squared (ε^2^) were reported as effect sizes. Significant global tests were followed by pairwise Mann–Whitney *U* tests with Holm adjustment. Between-group comparisons were considered exploratory and were not adjusted across outcomes.

Multinomial logistic regression was used to examine factors associated with following a self-identified vegan or vegetarian rather than an omnivorous dietary pattern. Omnivorous women constituted the reference outcome group. The primary model included age group, BMI, elevated eating disorder risk, healthy orthorexia, and orthorexia nervosa. The model was designed to adjust for age and BMI while simultaneously estimating the independent associations of the three primary eating-related constructs: elevated eating disorder risk, healthy orthorexia, and orthorexia nervosa. Variables were not selected based on univariate statistical significance. An additional sensitivity analysis included educational level to evaluate the robustness of the orthorexia findings to further sociodemographic adjustment. Age was entered categorically, with 18–24 years as the reference category, whereas BMI and both orthorexia scores were entered continuously. No multiplicity correction was applied to coefficients in the primary model.

Sensitivity analyses used the continuous EAT-26 score, restricted the sample to participants aged 25–44 years, and modeled age continuously using a restricted cubic spline, with knots at 19, 24, and 41 years, while retaining the spline specification for orthorexia nervosa. As secondary exploratory analyses, the nine food choice motives were examined in separate models by adding each subscale individually to the primary model. Two-degree-of-freedom joint Wald-test *p* values were adjusted using the Benjamini–Hochberg procedure, and contrast-specific *p* values within each motive were adjusted using Holm’s procedure. Contrast-specific findings were interpreted only when the corresponding joint test remained significant after adjustment.

Results were reported as adjusted odds ratios with 95% confidence intervals. Model fit was evaluated using the likelihood ratio test and Cox–Snell and Nagelkerke pseudo-R^2^ statistics. The linearity of the logit was assessed using the Box–Tidwell procedure across four continuous predictors and two outcome contrasts, with a Bonferroni-adjusted significance threshold of *p* < 0.00625. Because the Box–Tidwell assessment indicated nonlinearity for orthorexia nervosa in the vegan versus omnivorous contrast, orthorexia nervosa was modeled using a restricted cubic spline with three knots at the 10th, 50th, and 90th percentiles (scores of 0, 3, and 9). Three knots were used to limit model complexity given the sparse observations at higher scores. Model fit was compared with the linear model using a likelihood ratio test, and contrast-specific nonlinear components were evaluated using Wald tests. Covariate-adjusted marginal predicted probabilities with 95% confidence intervals were estimated across the observed score range. Because orthorexia nervosa scores included zero values, a constant of 1 was added solely when constructing its logarithmic interaction term. Multicollinearity was assessed using variance inflation factors. All tests were two-sided, with *p* < 0.05 considered statistically significant unless otherwise specified. Analyses were performed using IBM SPSS Statistics 30.0 (IBM Corp., Armonk, NY, USA) and Python 3.12.13 (Python Software Foundation, Wilmington, DE, USA).

## 3. Results

### 3.1. Participant Characteristics

The final analytical sample comprised 305 women, including 64 (21.0%) vegan, 71 (23.3%) vegetarian, and 170 (55.7%) omnivorous participants. As shown in Table 1, age group distribution differed across dietary patterns, χ^2^(6) = 70.561, *p* < 0.001, Cramér’s V = 0.340. An exploratory difference was also observed in elevated eating disorder risk, χ^2^(2) = 6.304, *p* = 0.043, although the association was small (Cramér’s V = 0.144). Educational level and BMI category did not differ across dietary patterns.

### 3.2. Age, BMI, Eating-Related Measures, and Food Choice Motives Across Dietary Patterns

Group comparisons are presented in Table 2. Vegan and vegetarian women were older than omnivorous women (*H* = 46.941, *p* < 0.001, ε^2^ = 0.149), whereas BMI and EAT-26 scores did not differ across dietary patterns. Vegan women had higher healthy orthorexia scores than omnivorous women (*H* = 16.474, *p* < 0.001, ε^2^ = 0.048) and lower orthorexia nervosa scores than both vegetarian and omnivorous women (*H* = 10.936, *p* = 0.004, ε^2^ = 0.030).

Holm-adjusted pairwise comparisons showed that vegan women assigned greater importance to natural content and less importance to weight control than omnivorous women. Omnivorous women assigned greater importance to familiarity than both vegan and vegetarian women and greater importance to ethical concern than vegetarian women. Although the global test for mood was significant, no pairwise comparison remained significant after adjustment. Among outcomes with significant global differences, effect sizes were small (ε^2^ = 0.015–0.048), except for age.

### 3.3. Multinomial Logistic Regression Analyses

The primary multinomial logistic regression results are presented in Table 3. The model was statistically significant, LR χ^2^(14) = 102.124, *p* < 0.001, with Cox–Snell and Nagelkerke pseudo–R^2^ values of 0.285 and 0.330, respectively. Compared with women aged 18–24 years, those aged 25–34 and 35–44 years had higher adjusted odds of following either a vegan or vegetarian rather than an omnivorous dietary pattern (Table 3).

Higher healthy orthorexia scores were associated with greater adjusted odds of following a vegan rather than an omnivorous dietary pattern (aOR = 1.177, 95% CI: 1.090–1.270, *p* < 0.001), whereas higher orthorexia nervosa scores were associated with lower adjusted odds (aOR = 0.827, 95% CI: 0.721–0.948, *p* = 0.007). Neither orthorexia dimension was associated with the vegetarian dietary pattern. BMI and elevated eating disorder risk were not associated with either dietary pattern.

In the secondary exploratory analyses, each food choice motive was examined in a separate adjusted model. After false discovery rate correction across the nine models, only weight control and familiarity showed significant joint associations with dietary pattern. Greater importance assigned to weight control was associated with lower odds of following either a vegan (aOR = 0.424, 95% CI: 0.249–0.723, Holm-adjusted *p* = 0.003) or vegetarian dietary pattern (aOR = 0.610, 95% CI: 0.382–0.975, Holm-adjusted *p* = 0.039). Greater importance assigned to familiarity was associated with lower odds of following a vegan dietary pattern (aOR = 0.466, 95% CI: 0.296–0.735, Holm-adjusted *p* = 0.002), whereas the corresponding association with the vegetarian pattern was not significant after adjustment. No other food choice motive had a significant joint association after false discovery rate adjustment.

### 3.4. Sensitivity Analyses

Box–Tidwell tests indicated nonlinearity for continuous age and orthorexia nervosa in the vegan versus omnivorous contrast. Age was therefore modeled categorically, and the orthorexia nervosa association was further examined in a quadratic sensitivity analysis. No problematic multicollinearity was detected (all variance inflation factors ≤1.54).

Replacing elevated eating disorder risk with the continuous EAT-26 score did not materially change the primary model results. EAT-26 scores were not associated with either the vegan (aOR = 0.983, 95% CI: 0.942–1.026, *p* = 0.432) or vegetarian dietary pattern (aOR = 0.985, 95% CI: 0.954–1.017, *p* = 0.352), whereas the associations of both orthorexia dimensions with the vegan pattern were retained (Supplementary Table S2).

**Figure 1:**
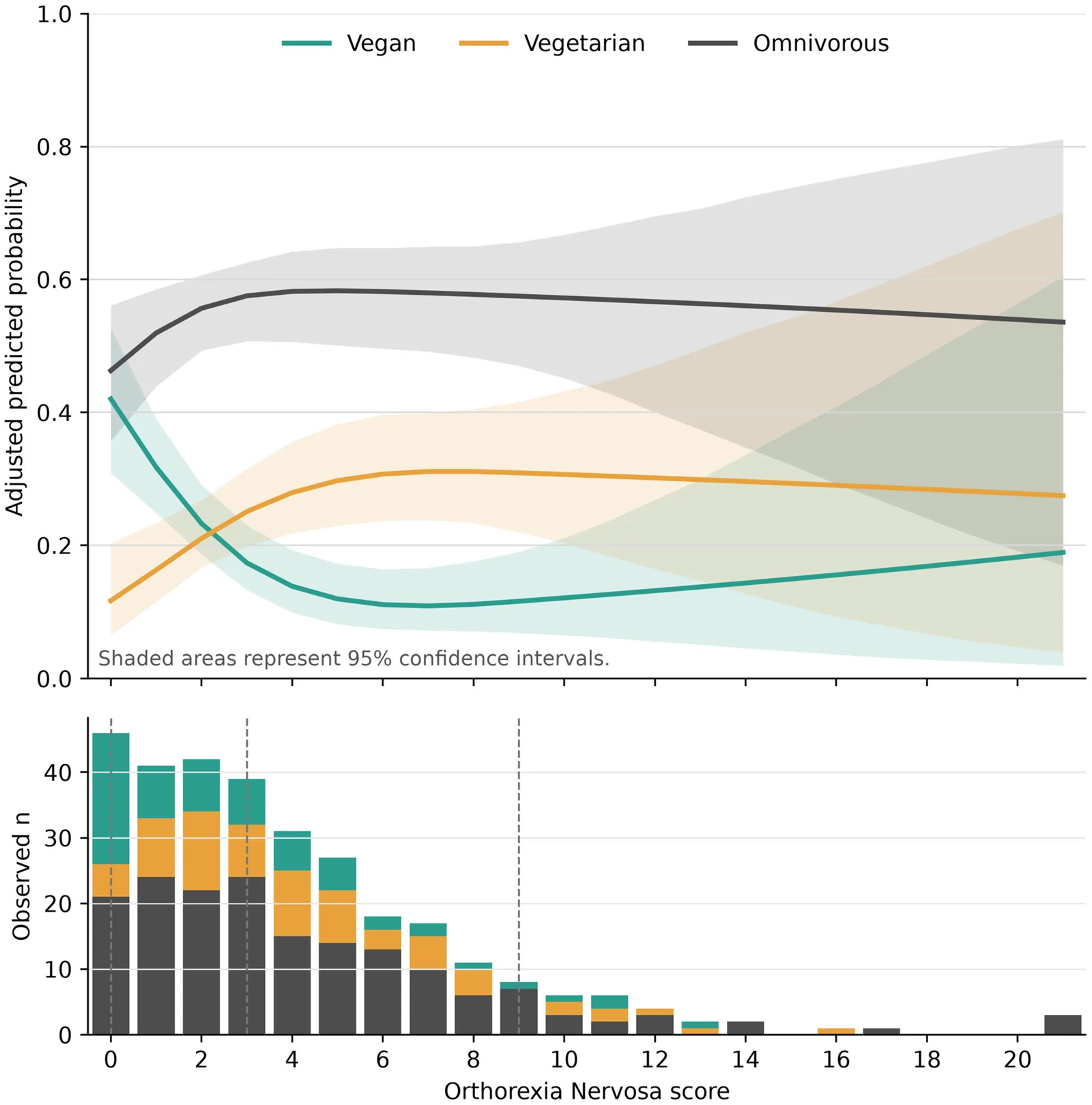
Adjusted predicted probabilities of dietary patterns across orthorexia nervosa scores. Probabilities were estimated from the restricted cubic spline multinomial logistic regression model adjusted for age group, BMI, elevated eating disorder risk, and healthy orthorexia. Shaded areas represent 95% confidence intervals. The lower panel shows the observed score distribution by dietary pattern. Dashed vertical lines indicate spline knots at scores of 0, 3, and 9.

The restricted cubic spline model provided a better fit than the corresponding linear model, LR χ^2^(2) = 9.619, *p* = 0.008. The nonlinear component was significant for the vegan versus omnivorous contrast, Wald χ^2^(1) = 7.568, *p* = 0.006, but not for the vegetarian versus omnivorous contrast, Wald χ^2^(1) = 0.594, *p* = 0.441. As shown in Figure 1, the adjusted probability of following a vegan dietary pattern decreased from 0.420 at an orthorexia nervosa score of 0 to 0.174 at a score of 3 and 0.111 at a score of 6, after which the curve flattened. Estimates became increasingly imprecise at higher scores, where observations were sparse; only 19 participants had scores ≥11 and nine had scores ≥13. Therefore, the linear odds ratio should not be interpreted as constant across the observed score range (Supplementary Table S3).

In the analysis restricted to participants aged 25–44 years (*n* = 123), higher healthy orthorexia scores remained associated with greater odds of following a vegan rather than an omnivorous dietary pattern (aOR = 1.203, 95% CI: 1.079–1.341, *p* = 0.001), whereas higher orthorexia nervosa scores remained associated with lower odds (aOR = 0.777, 95% CI: 0.635–0.952, *p* = 0.015). Neither dimension was associated with the vegetarian dietary pattern (Supplementary Table S4).

When age was modeled continuously using a restricted cubic spline, the substantive findings were unchanged. Healthy orthorexia remained associated with greater odds of following a vegan rather than an omnivorous dietary pattern (aOR = 1.197, 95% CI: 1.107–1.295, *p* < 0.001). The overall association between orthorexia nervosa and the vegan pattern remained significant, Wald χ^2^(2) = 14.486, *p* = 0.001, and its nonlinear component was retained, Wald χ^2^(1) = 8.579, *p* = 0.003. Neither orthorexia dimension was associated with the vegetarian pattern (Supplementary Table S5).

Further adjustment for educational level did not materially alter the findings. Education was not jointly associated with either the vegan versus omnivorous contrast (Wald χ^2^(3) = 1.259, *p* = 0.739) or the vegetarian versus omnivorous contrast (Wald χ^2^(3) = 1.118, *p* = 0.773). Healthy orthorexia remained associated with the vegan pattern (aOR = 1.191, 95% CI: 1.100–1.290, *p* < 0.001), and the nonlinear component of orthorexia nervosa remained significant for the vegan versus omnivorous contrast (Wald χ^2^(1) = 8.209, *p* = 0.004). Neither orthorexia dimension was associated with the vegetarian pattern (Supplementary Table S6).

## 4. Discussion

This study examined eating disorder risk, healthy orthorexia, and orthorexia nervosa across self-identified vegan, vegetarian, and omnivorous dietary patterns. In the adjusted model, elevated eating disorder risk was not significantly associated with either the vegan or vegetarian pattern. Higher healthy orthorexia scores were associated with the vegan pattern, whereas the association between orthorexia nervosa and the vegan pattern was nonlinear. Neither orthorexia dimension was associated with the vegetarian pattern. In the secondary exploratory analyses, weight control and familiarity were the only food choice motives that remained associated with dietary pattern after adjustment and correction for multiple testing.

No statistically significant adjusted association was detected between elevated eating disorder risk and either dietary pattern. However, only four vegan and ten vegetarian participants met the EAT-26 risk threshold, and the corresponding estimates were imprecise, particularly for the vegan versus omnivorous contrast (aOR = 0.471, 95% CI: 0.133–1.669). Therefore, these findings should not be interpreted as demonstrating equivalence or the absence of a clinically meaningful association. The sensitivity analysis using continuous EAT-26 scores produced a similar pattern of results but did not eliminate this limitation. This finding is consistent with a recent meta-analysis reporting no overall difference in eating disorder symptoms between adults following vegan or vegetarian diets and those following omnivorous diets [10], as well as with findings by Norwood et al. [9] and Dorard and Mathieu [20]. Nevertheless, a systematic review by Mathieu et al. [27] found heterogeneous associations between vegetarianism and disordered eating, depending on the sample and eating-related dimension examined. Evidence from clinical and retrospective samples is more complex. Bardone-Cone et al. [5], using a broad definition that included partial meat avoidance, found that women with an eating disorder history reported vegetarianism more frequently and that eating disorder symptoms often preceded dietary restriction. Differences in clinical status, sampling, dietary definitions, motivations, and assessment instruments may contribute to these divergent findings. Interpretation of the present results also requires consideration of the EAT-26 itself. McLean et al. [28] reported inadequate fit for commonly proposed factor structures and generally poor test–retest reliability among vegetarian and vegan samples, raising questions about the instrument’s stability in these populations. Moreover, conventional measures may not adequately distinguish restriction motivated by weight or shape concerns from food exclusions inherent in vegan or vegetarian diets [4]. These findings suggest that eating disorder risk should be assessed individually rather than inferred from dietary pattern labels alone.

Healthy orthorexia and orthorexia nervosa showed contrasting associations with the vegan dietary pattern. Consistent with this distinction, Albery et al. [18] found higher healthy orthorexia among vegan and vegetarian participants than among omnivores, but no dietary group differences in orthorexia nervosa. Research conducted in Türkiye has also examined the two dimensions separately among vegetarians and omnivores, providing relevant evidence within the present sociocultural context [19]. In contrast, Şentürk et al. [16] reported higher healthy orthorexia among vegan participants together with higher orthorexia nervosa among vegan and vegetarian participants, while a recent meta-analysis found higher orthorexia nervosa symptoms among adults following vegan or vegetarian diets [17]. Differences in sampling, dietary definitions, assessment instruments, and covariate adjustment may contribute to these divergent findings. Findings obtained using instruments that do not adequately distinguish a non-pathological interest in healthy eating from pathological preoccupation and impairment should be interpreted cautiously [4,14,29]. The restricted cubic spline analysis showed that the association between orthorexia nervosa and the vegan dietary pattern was nonlinear. The adjusted probability of following a vegan pattern declined markedly across low-to-moderate scores but flattened at higher scores. Estimates in the upper score range were imprecise because observations were sparse. Therefore, the linear odds ratio should not be interpreted as constant across the scale or as evidence that veganism protects against orthorexia nervosa. Although the TOS has been validated in Turkish, its measurement equivalence across dietary pattern groups has not been established, and the conceptual boundary between orthorexia nervosa and broader eating pathology remains debated. Overall, these findings support assessing healthy orthorexia and orthorexia nervosa as distinct dimensions.

In the secondary exploratory analyses, weight control and familiarity were the only food choice motives that remained associated with dietary pattern after adjustment and correction for multiple testing. The lower importance assigned to weight control among vegan and vegetarian women is broadly consistent with findings by Dorard and Mathieu [20], Kim et al. [30], and Hanras et al. [21]. However, Forestell et al. [31] found no difference between vegetarian and omnivorous women and reported greater weight control motivation among semi-vegetarian and flexitarian women. These differences may reflect variation in dietary group definitions and the degree of animal product restriction. Although previous research has linked weight control motivation more closely to orthorexia nervosa than to healthy orthorexia [32], the present findings do not establish that lower weight control motivation and the two orthorexia dimensions represent a shared individual profile. These secondary findings should be considered exploratory, particularly the familiarity finding, because this subscale had internal consistency below 0.70 and the food choice motives were examined in separate rather than mutually adjusted models.

The lower importance assigned to familiarity among vegan women is consistent with previous findings that omnivorous individuals place greater importance on familiar foods than vegetarians and vegans [21,31]. Forestell et al. [31] also reported lower food neophobia and greater openness to experience and variety seeking among vegetarian women. Reduced reliance on familiarity may therefore reflect greater willingness to consider foods outside conventional dietary practices, although these characteristics were not directly assessed in the present study. Moreover, current food choice motives should not be interpreted as reasons for initially adopting a dietary pattern, as such motives may change over time and across sociocultural contexts [33]. The cross-sectional design precludes determining whether lower familiarity motivation preceded adoption of the vegan pattern or developed during continued adherence.

The principal strengths of this study were the separate evaluation of vegan and vegetarian dietary patterns and the use of the Teruel Orthorexia Scale to distinguish healthy orthorexia from orthorexia nervosa. Eating disorder risk, both orthorexia dimensions, and food choice motives were evaluated within the same sample using validated Turkish instruments. The adjusted analyses, sensitivity analyses addressing key modeling decisions, and multiplicity correction applied to the secondary food choice motive models further strengthened the interpretation of the findings.

Several limitations should be considered. The cross-sectional design precludes causal inference, while convenience sampling through social media, inclusion of women only, and reliance on self-reported data limit generalizability. The dietary groups also differed substantially in age, with women aged 18–24 years disproportionately represented in the omnivorous group. Although age was included in the adjusted analyses, the limited age overlap across the dietary groups may have reduced the comparability of the groups and contributed to unstable estimates or residual confounding. Therefore, the observed age associations should be interpreted cautiously and should not be generalized to the broader population of women in Türkiye. No formal a priori sample size calculation was performed, and the unequal and relatively small vegan and vegetarian groups may have limited estimate precision, particularly because few participants met the EAT-26 risk threshold. Dietary patterns were self-identified and not verified through dietary assessment, potentially resulting in exposure misclassification. Duration of adherence and reasons for dietary pattern adoption were also not assessed. Previous dieting or weight control history, mental health characteristics, socioeconomic status, and physical activity were also not assessed and may have contributed to residual confounding. Nutritional knowledge was likewise not assessed and may have contributed to residual confounding, as recent evidence from a large Turkish adult sample indicates associations with both eating disorder symptoms and orthorexia-related scores [34]. Measurement equivalence across dietary groups was not examined, and the internal consistency coefficients of the familiarity and price subscales were below 0.70, warranting caution in interpreting the familiarity finding. Finally, food choice motives were examined in separate models and were therefore not mutually adjusted. Accordingly, the findings should not be generalized to the broader population of women in Türkiye.

## 5. Conclusions

In this convenience sample of women, no statistically significant adjusted association was detected between elevated eating disorder risk and either the vegan or vegetarian dietary pattern; however, the limited number of participants meeting the EAT-26 risk threshold precludes concluding that no clinically meaningful association exists. Higher healthy orthorexia scores were associated with the vegan pattern, whereas the association between orthorexia nervosa and the vegan pattern was nonlinear and estimated imprecisely at higher scores. Neither orthorexia dimension was associated with the vegetarian pattern. These findings emphasize the importance of distinguishing a non-pathological interest in healthy eating from pathological rigidity and impairment. Secondary exploratory analyses also identified lower weight control motivation in both vegan and vegetarian women and lower familiarity motivation in vegan women. Clinically, vegan or vegetarian dietary pattern labels should neither be treated as indicators of eating pathology nor assumed to exclude it. Assessment should instead consider the motivations underlying food exclusions, weight- and shape-related concerns, dietary rigidity, distress, and functional impairment. When concerns are identified, collaborative assessment by dietitians and mental health professionals may be appropriate. Given the cross-sectional design and convenience sample, these clinical implications should be considered provisional and require confirmation in more representative longitudinal studies. Longitudinal studies using detailed dietary assessment and measures validated across dietary groups are needed to clarify the temporal and clinical significance of these associations.

## Figures and Tables

**Table 1:** Categorical characteristics of women according to self-identified dietary pattern.

Characteristic	Category	Vegan (*n* = 64)	Vegetarian (*n* = 71)	Omnivorous (*n* = 170)	χ^2^ (df)	*p*	Cramér’s V
Age group	18–24 years	16 (25.0)	21 (29.6)	123 (72.4)	70.561 (6)	**<0.001**	0.340
	25–34 years	30 (46.9)	36 (50.7)	24 (14.1)			
	35–44 years	14 (21.9)	8 (11.3)	11 (6.5)			
	≥45 years	4 (6.2)	6 (8.5)	12 (7.1)			
Education	High school	7 (10.9)	4 (5.6)	11 (6.5)	12.089 (6)	0.060 ^a^	0.141
	Bachelor’s degree	38 (59.4)	48 (67.6)	135 (79.4)			
	Master’s degree	13 (20.3)	14 (19.7)	17 (10.0)			
	Doctorate	6 (9.4)	5 (7.0)	7 (4.1)			
BMI category	Underweight	5 (7.8)	5 (7.0)	18 (10.6)	1.920 (6)	0.927	0.056
	Normal weight	44 (68.8)	50 (70.4)	120 (70.6)			
	Overweight	12 (18.8)	14 (19.7)	26 (15.3)			
	Obesity	3 (4.7)	2 (2.8)	6 (3.5)			
Eating disorder risk	Present	4 (6.2)	10 (14.1)	33 (19.4)	6.304 (2)	**0.043**	0.144
	Absent	60 (93.8)	61 (85.9)	137 (80.6)			

Note. Values are *n* (%), with percentages calculated within each dietary pattern group. *p* values were obtained using Pearson’s chi-square tests and were not adjusted across outcomes. Cramér’s V represents effect size. Statistically significant *p* values are shown in bold. ^a^ The *p* value for educational level was estimated using a Monte Carlo procedure because more than 20% of cells had expected counts below five.

**Table 2:** Age, BMI, eating-related measures, and food choice motives according to dietary pattern.

Outcome	Total Sample (*N* = 305)	Vegan (*n* = 64)	Vegetarian (*n* = 71)	Omnivorous (*n* = 170)	*H*	*p*	ε^2^	Holm Adjusted Post Hoc
Age, years	24.00 (20.00–31.00)	29.00 (24.75–36.00)	28.00 (23.00–32.50)	21.00 (20.00–25.00)	46.941	**<0.001**	0.149	V > O; Veg > O
BMI, kg/m^2^	21.26 (19.53–23.92)	21.34 (19.48–24.93)	21.26 (19.78–23.57)	21.24 (19.49–23.65)	0.356	0.837	0.000	-
Healthy orthorexia	14.00 (10.00–18.00)	17.00 (12.00–20.00)	15.00 (11.50–18.00)	13.00 (10.00–17.00)	16.474	**<0.001**	0.048	V > O
Orthorexia nervosa	3.00 (1.00–6.00)	2.00 (0.00–4.00)	4.00 (2.00–6.00)	3.00 (1.00–6.00)	10.936	**0.004**	0.030	V < Veg; V < O
EAT-26	8.00 (4.00–15.00)	7.00 (3.00–13.25)	7.00 (3.00–14.00)	9.00 (4.00–16.00)	4.916	0.086	0.010	-
Health	3.00 (2.50–3.50)	3.08 (2.67–3.67)	3.00 (2.33–3.33)	3.00 (2.37–3.33)	3.951	0.139	0.006	-
Mood	2.83 (2.17–3.33)	2.67 (2.00–3.17)	2.67 (2.08–3.00)	2.83 (2.33–3.33)	7.698	**0.021**	0.019	-
Convenience	3.00 (2.40–3.60)	3.00 (2.55–3.40)	3.20 (2.60–3.60)	2.80 (2.40–3.40)	5.080	0.079	0.010	-
Natural content	3.00 (2.33–3.33)	3.00 (2.33–3.67)	3.00 (2.33–3.33)	2.67 (2.08–3.33)	8.782	**0.012**	0.022	V > O
Price	3.00 (2.33–3.33)	3.00 (2.67–3.33)	3.00 (2.33–3.33)	3.00 (2.33–3.33)	0.163	0.922	0.000	-
Weight control	2.33 (1.67–3.00)	2.00 (1.67–2.67)	2.33 (1.67–2.67)	2.33 (2.00–3.00)	6.524	**0.038**	0.015	V < O
Sensory appeal	3.25 (2.75–3.75)	3.00 (2.69–3.50)	3.00 (2.50–3.50)	3.25 (2.75–3.75)	3.383	0.184	0.005	-
Familiarity	2.67 (2.00–3.00)	2.33 (1.67–3.00)	2.33 (2.00–3.00)	2.67 (2.33–3.33)	16.450	**<0.001**	0.048	O > V; O > Veg
Ethical concern	2.33 (1.67–3.00)	2.33 (1.67–3.00)	2.00 (1.33–2.67)	2.33 (1.67–3.00)	7.102	**0.029**	0.017	Veg < O

Note. Values are medians (interquartile ranges). Global comparisons were performed using Kruskal–Wallis tests. Significant global tests were followed by pairwise Mann–Whitney *U* tests with Holm adjustment; only significant adjusted comparisons are shown. Between-group comparisons were considered exploratory and were not adjusted across outcomes. Negative epsilon-squared estimates were reported as zero. Statistically significant *p* values are shown in bold. ε^2^, epsilon-squared; V, vegan; Veg, vegetarian; O, omnivorous.

**Table 3:** Multinomial logistic regression results for the primary model and secondary exploratory food choice motive models.

Panel A. Primary Model										
Predictor		Vegan vs. Omnivorous aOR (95% CI)			*p*	Vegetarian vs. Omnivorous aOR (95% CI)			*p*	
**Age 25–34 vs.** **18–24 years**		7.094 (3.231–15.579)			**<0.001**	9.115 (4.433–18.742)			**<0.001**	
**Age 35–44 vs.** **18–24 years**		5.954 (2.141–16.557)			**0.001**	3.453 (1.199–9.949)			**0.022**	
**Age ≥ 45 vs.** **18–24 years**		1.427 (0.383–5.326)			0.596	2.663 (0.832–8.520)			0.099	
**BMI, per kg/m^2^**		1.064 (0.972–1.164)			0.179	1.033 (0.948–1.126)			0.460	
**Eating disorder risk**		0.471 (0.133–1.669)			0.243	0.715 (0.281–1.821)			0.482	
**Healthy orthorexia**		1.177 (1.090–1.270)			**<0.001**	1.041 (0.976–1.111)			0.222	
**Orthorexia nervosa**		0.827 (0.721–0.948)			**0.007**	1.045 (0.950–1.149)			0.362	
**Panel B. Secondary Exploratory Food Choice Motive Models**										
**Food** **C** **hoice ** **M** **otive**	**Vegan vs** **. O** **mnivorous** **aOR (95% CI)**		**Holm-** **A** **djusted *p***	**Vegetarian vs** **. O** **mnivorous** **aOR (95% CI)**			**Holm-** **A** **djusted *p***	**Joint *p***		**FDR *q***
**Health**	1.392 (0.805–2.404)		0.472	1.001 (0.623–1.607)			0.997	0.452		0.625
**Mood**	0.860 (0.544–1.359)		0.518	0.767 (0.506–1.161)			0.419	0.445		0.625
**Convenience**	1.385 (0.849–2.260)		0.192	1.474 (0.946–2.297)			0.172	0.173		0.390
**Natural content**	1.097 (0.661–1.820)		1.000	0.984 (0.640–1.513)			1.000	0.918		0.918
**Price**	1.273 (0.779–2.079)		0.672	1.206 (0.776–1.874)			0.672	0.556		0.625
**Weight control**	0.424 (0.249–0.723)		**0** **.003**	0.610 (0.382–0.975)			**0** **.039**	**0** **.004**		**0** **.020**
**Sensory appeal**	0.810 (0.486–1.350)		0.607	0.794 (0.511–1.233)			0.607	0.529		0.625
**Familiarity**	0.466 (0.296–0.735)		**0** **.002**	0.683 (0.452–1.034)			0.071	**0** **.004**		**0** **.020**
**Ethical concern**	1.045 (0.673–1.624)		0.844	0.620 (0.413–0.933)			**0** **.044**	**0** **.043**		0.130

Note. Omnivorous women constituted the reference outcome group. Panel A presents the primary model, which included age group, BMI, elevated eating disorder risk, healthy orthorexia, and orthorexia nervosa simultaneously. Panel B presents secondary exploratory models in which each food choice motive was added separately to the primary model. Joint *p* values were obtained from two-degree-of-freedom Wald tests, and FDR *q* values were calculated using the Benjamini–Hochberg procedure across the nine motives. Contrast-specific *p* values were Holm-adjusted within each motive. The 95% confidence intervals were not adjusted for multiplicity. Statistically significant *p* values are shown in bold. aOR, adjusted odds ratio; CI, confidence interval; BMI, body mass index; FDR, false discovery rate.

## Data Availability

The data supporting the findings of this study are available from the corresponding author upon reasonable request. The data are not publicly available because they contain participant information subject to ethical and privacy considerations.

## Supplementary Materials

The following supporting information can be downloaded at: https://www.mdpi.com/article/10.3390/healthcare14182914/s1, Table S1: Internal consistency of the study measures; Table S2: Sensitivity analysis using the continuous EAT-26 total score in the multinomial logistic regression model; Table S3: Restricted cubic spline analysis of the association between orthorexia nervosa and dietary pattern; Table S4: Sensitivity analysis restricted to participants aged 25–44 years; Table S5: Sensitivity analysis modeling age continuously using a restricted cubic spline; Table S6: Sensitivity analysis additionally adjusted for educational level.
