# Supplementary material for: Eating Disorder Risk, Healthy Orthorexia, and Orthorexia Nervosa Among Women Following Vegan, Vegetarian, and Omnivorous Dietary Patterns in Türkiye: A Cross-Sectional Study
Source: Healthcare (Basel). 2026 Sep 9;14(18):2914. doi: 10.3390/healthcare14182914 (PMC13606553; doi:10.3390/healthcare14182914)
Supplement: Supplementary file 1 [file healthcare-14-02914-s001.zip › healthcare-4512297-supplementary.pdf]

Supplementary Materials

Eating-Disorder Risk, Healthy Orthorexia, and Orthorexia Nervosa among Women Following Vegan, Vegetarian, and Omnivorous Dietary Patterns in Türkiye: A Cross-Sectional Study

Supplementary Table S1. Internal consistency of the study measures

Scale or subscale	Number of items	Cronbach's α
EAT-26	26	.892
TOS—Healthy Orthorexia	9	.831
TOS—Orthorexia Nervosa	7	.802
FCQ—Health	6	.875
FCQ—Mood	6	.870
FCQ—Convenience	5	.827
FCQ—Natural content	3	.831
FCQ—Price	3	.688
FCQ—Weight control	3	.731
FCQ—Sensory appeal	4	.792
FCQ—Familiarity	3	.686
FCQ—Ethical concern	3	.748

Note. Cronbach's alpha coefficients were calculated in the full sample (N = 305). EAT-26, Eating Attitudes Test-26; TOS, Teruel Orthorexia Scale; FCQ, Food Choice Questionnaire.

Supplementary Table S2. Sensitivity analysis using the continuous EAT-26 total score in the multinomial logistic regression model

Predictor	Vegan vs omnivorous aOR (95% CI)	p	Vegetarian vs omnivorous aOR (95% CI)	p
Age 25–34 vs 18–24 years	6.960 (3.161–15.321)	**<.001**	8.912 (4.326–18.358)	**<.001**
Age 35–44 vs 18–24 years	5.859 (2.097–16.376)	**.001**	3.351 (1.159–9.690)	**.026**
Age ≥45 vs 18–24 years	1.319 (0.354–4.909)	.680	2.567 (0.800–8.236)	.113
BMI, per kg/m²	1.064 (0.971–1.165)	.183	1.035 (0.949–1.129)	.435
EAT-26 total score, per point	0.983 (0.942–1.026)	.432	0.985 (0.954–1.017)	.352
Healthy Orthorexia	1.178 (1.091–1.272)	**<.001**	1.042 (0.976–1.113)	.214
Orthorexia Nervosa	0.824 (0.717–0.946)	**.006**	1.054 (0.954–1.164)	.302

Note. Omnivorous women constituted the reference outcome group. The model included age group, BMI, continuous EAT-26 total score, Healthy Orthorexia score, and Orthorexia Nervosa score. All predictors were entered simultaneously. aOR, adjusted odds ratio; CI, confidence interval; EAT-26, Eating Attitudes Test-26. Overall model: likelihood-ratio $\chi^2(14) = 101.750$, $p < .001$; Nagelkerke pseudo-$R^2 = .329$. Statistically significant p values are shown in bold.

Supplementary Table S3. Restricted cubic spline analysis of the association between Orthorexia Nervosa and dietary pattern

**Analysis**	**Contrast**	**χ^2**	**df**	**p**
Spline versus linear model	Overall model comparison	9.619	2	**.008**
Overall Orthorexia Nervosa association	Vegan versus omnivorous	13.003	2	**.002**
Nonlinear component	Vegan versus omnivorous	7.568	1	**.006**
Overall Orthorexia Nervosa association	Vegetarian versus omnivorous	1.316	2	.518
Nonlinear component	Vegetarian versus omnivorous	0.594	1	.441

Note. Omnivorous women constituted the reference outcome group. The model was adjusted for age group, BMI, elevated eating-disorder risk, and Healthy Orthorexia. Restricted cubic spline knots were placed at Orthorexia Nervosa scores of 0, 3, and 9. Model comparison used a likelihood-ratio test; contrast-specific tests used Wald tests. Statistically significant p values are shown in bold.

Supplementary Table S4. Sensitivity analysis restricted to participants aged 25–44 years

Predictor	Vegan vs omnivorous aOR (95% CI)	p	Vegetarian vs omnivorous aOR (95% CI)	p
Age 35–44 vs 25–34 years	0.898 (0.296–2.721)	.849	0.409 (0.129–1.295)	.129
BMI, per kg/m²	1.002 (0.884–1.135)	.977	0.952 (0.834–1.086)	.464
Eating-disorder risk	0.535 (0.098–2.911)	.469	0.257 (0.041–1.594)	.144
Healthy Orthorexia	1.203 (1.079–1.341)	**.001**	1.077 (0.976–1.189)	.140
Orthorexia Nervosa	0.777 (0.635–0.952)	**.015**	0.983 (0.824–1.173)	.851

Note. The analysis was restricted to 123 participants aged 25–44 years, including 44 vegan, 44 vegetarian, and 35 omnivorous women. Omnivorous women constituted the reference outcome group. Women aged 25–34 years constituted the reference category for age. The model included age group, BMI, eating-disorder risk, Healthy Orthorexia, and Orthorexia Nervosa simultaneously. Statistically significant p values are shown in bold. aOR, adjusted odds ratio; CI, confidence interval; BMI, body mass index.

Supplementary Table S5. Sensitivity analysis modeling age continuously using a restricted cubic spline

**Panel A. Overall and nonlinear spline tests**				
**Predictor/test**	**Contrast**	**χ^2**	**df**	**p**
Overall age association	Vegan versus omnivorous	25.978	2	**<.001**
Nonlinear age component	Vegan versus omnivorous	20.149	1	**<.001**
Overall age association	Vegetarian versus omnivorous	34.877	2	**<.001**
Nonlinear age component	Vegetarian versus omnivorous	28.032	1	**<.001**
Overall Orthorexia Nervosa association	Vegan versus omnivorous	14.486	2	**.001**
Nonlinear Orthorexia Nervosa component	Vegan versus omnivorous	8.579	1	**.003**
Overall Orthorexia Nervosa association	Vegetarian versus omnivorous	0.348	2	.840
Nonlinear Orthorexia Nervosa component	Vegetarian versus omnivorous	0.148	1	.700
**Panel B. Estimates for predictors modeled linearly**				
**Predictor**	**Vegan versus omnivorous aOR (95% CI)**	**p**	**Vegetarian versus omnivorous aOR (95% CI)**	**p**
BMI	1.060 (0.966–1.163)	.217	1.038 (0.952–1.132)	.399
Elevated eating-disorder risk	0.340 (0.089–1.298)	.114	0.771 (0.305–1.949)	.583
Healthy Orthorexia	1.197 (1.107–1.295)	**<.001**	1.038 (0.970–1.110)	.278

Note. Omnivorous women constituted the reference outcome group. Age was modeled using a restricted cubic spline with knots at 19, 24, and 41 years; Orthorexia Nervosa was modeled using knots at scores of 0, 3, and 9. The model was additionally adjusted for BMI, elevated eating-disorder risk, and Healthy Orthorexia. aOR, adjusted odds ratio; CI, confidence interval. Statistically significant p values are shown in bold.

Supplementary Table S6. Sensitivity analysis additionally adjusted for educational level

Contrast and variable	aOR (95% CI)	Wald χ^2	df	p-value
**Vegan versus omnivorous**				
Educational level, overall	—	1.259	3	.739
Healthy Orthorexia	1.191 (1.100–1.290)	—	1	**<.001**
Orthorexia Nervosa, overall	—	13.868	2	**.001**
Orthorexia Nervosa, nonlinear component	—	8.209	1	**.004**
**Vegetarian versus omnivorous**				
Educational level, overall	—	1.118	3	.773
Healthy Orthorexia	1.035 (0.967–1.107)	—	1	.319
Orthorexia Nervosa, overall	—	0.331	2	.847
Orthorexia Nervosa, nonlinear component	—	0.113	1	.737

Note. Omnivorous women constituted the reference outcome group. The model included age and Orthorexia Nervosa as restricted cubic splines, with knots at 19, 24, and 41 years for age and at scores of 0, 3, and 9 for Orthorexia Nervosa. The model was additionally adjusted for BMI, elevated eating-disorder risk, Healthy Orthorexia, and educational level. Bachelor's degree was the reference category for educational level. aOR, adjusted odds ratio; CI, confidence interval. Significant p-values are shown in bold.
